# P-1596. Identifying Candidates for Blood Culture among Hospitalized COVID-19 Patients: a Multicenter Study in South Korea

**DOI:** 10.1093/ofid/ofaf695.1775

**Published:** 2026-01-11

**Authors:** Sung un Shin, Doyoung Han, Minji Kim, Ahrang Lee, Hae Seong Jung, Uh Jin Kim, Seung-Ji Kang, Kyung-Hwa Park, Sook In Jung, Seong Eun Kim

**Affiliations:** Chonnam National University hospital, Kangju, Kwangju-jikhalsi, Republic of Korea; Chonnam National University Medical School, Dong-gu, Kwangju-jikhalsi, Republic of Korea; Chonnam National University Hospital, GwangJu, Kwangju-jikhalsi, Republic of Korea; Department of Infectious Diseases, Chonnam National University Medical School,Gwangju, Korea., Gwangju, Kwangju-jikhalsi, Republic of Korea; Chonnam National University Medical School, Dong-gu, Kwangju-jikhalsi, Republic of Korea; Chonnam National University Medical School, Dong-gu, Kwangju-jikhalsi, Republic of Korea; Infectious Diseases, Gwang-ju, Kwangju-jikhalsi, Republic of Korea; Chonnam National University Medical School, Dong-gu, Kwangju-jikhalsi, Republic of Korea; Chonnam National University Medical School, Dong-gu, Kwangju-jikhalsi, Republic of Korea; Chonnam National University Medical School, Dong-gu, Kwangju-jikhalsi, Republic of Korea

## Abstract

**Background:**

During the COVID-19 pandemic, distinguishing bloodstream infections (BSI) from viral illness was challenging, often leading to excessive culture use and increased diagnostic burden in referral hospitals. We aimed to identify clinical predictors of BSI in hospitalized COVID-19 patients to guide targeted diagnostics and optimize resource allocation.

**Figure 1.**
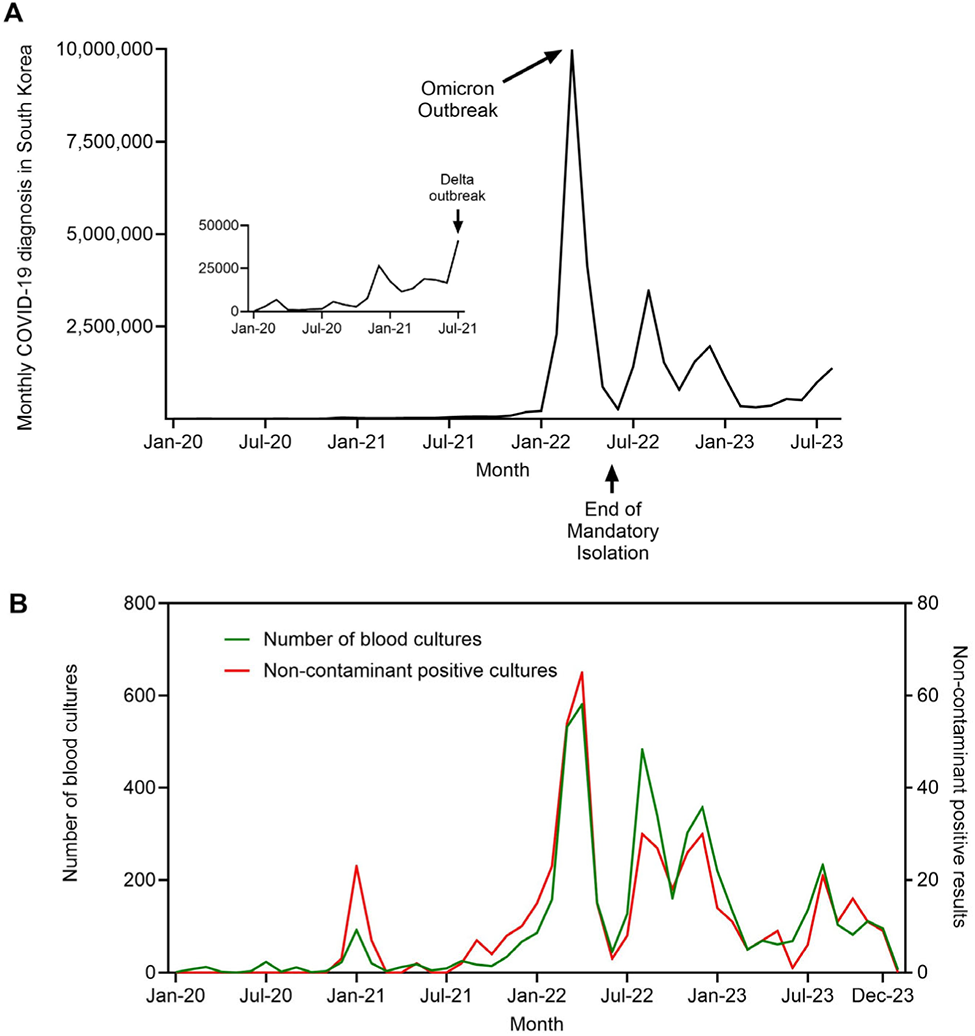
Temporal trends in COVID-19 cases, blood culture testing, and BSI during the pandemic in South Korea. (A) Monthly number of confirmed COVID-19 cases in South Korea from January 2020 to August 2023, with major outbreak periods (Delta and Omicron) indicated. The inset shows the early pandemic phase. (B) Monthly number of patients who underwent blood culture within 14 days of COVID-19 diagnosis (green), and those with non-contaminant bloodstream infections (red). Both blood culture utilization and BSI detection increased during outbreak peaks, reflecting the diagnostic burden associated with pandemic surges.

**Figure 2.**
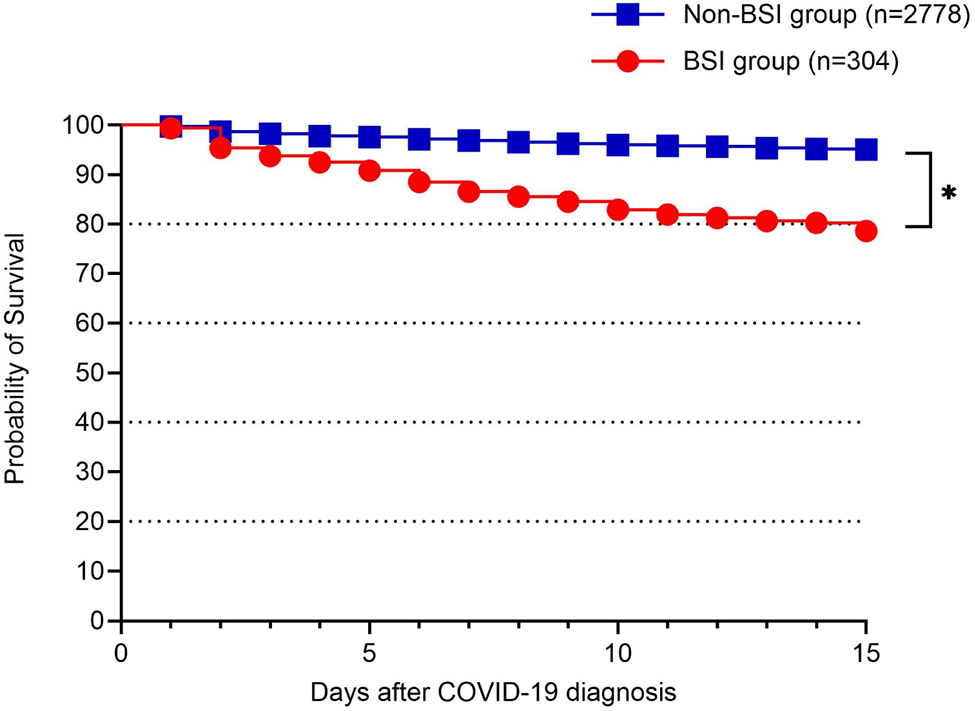
Kaplan–Meier survival analysis by blood culture result within 15 days of COVID-19 diagnosis Kaplan–Meier curves comparing 15-day survival between patients with non-contaminant bloodstream infection (red) and those with negative or contaminated cultures (blue) following COVID-19 diagnosis. Survival was significantly lower in the BSI group (P<0.001).

**Methods:**

We conducted a retrospective cohort study across three hospitals in South Korea (Jan 2020 - Dec 2023). Patients with confirmed COVID-19 who underwent blood culture within 15 days were included. BSI was defined as isolation of non-commensal organisms or commensals from ≥2 concurrent culture sets. Data were extracted from electronic records. The primary outcome was 15-day mortality. Independent predictors of BSI were identified via multivariate logistic regression.

**Figure:**
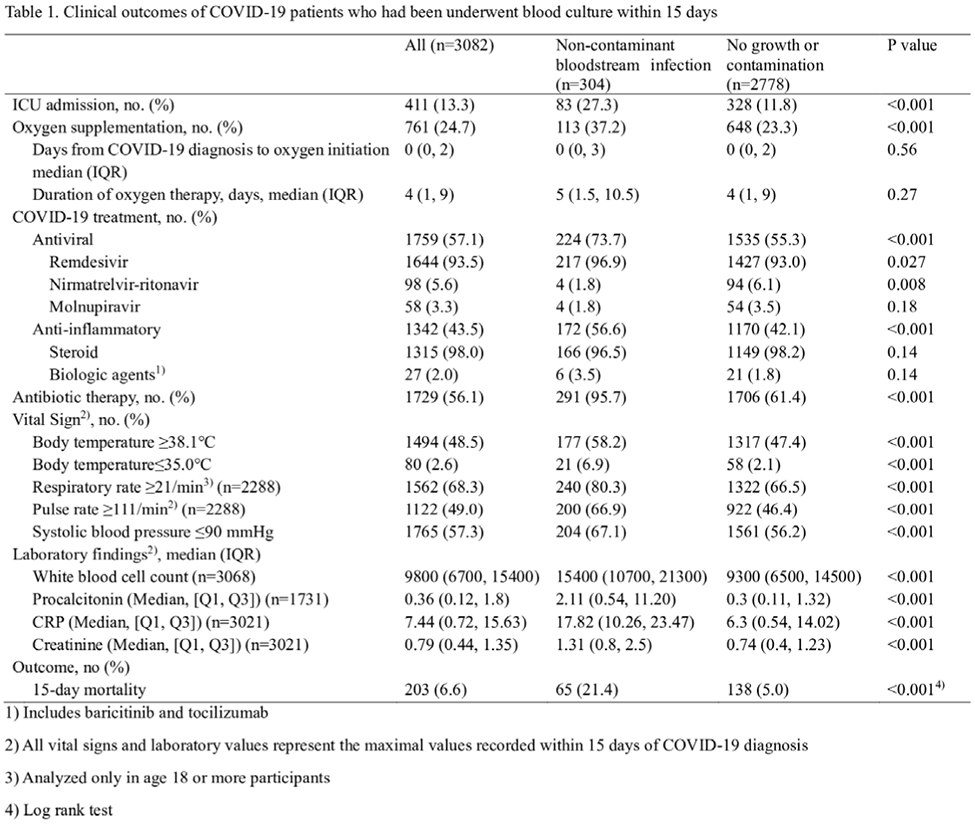

**Figure:**
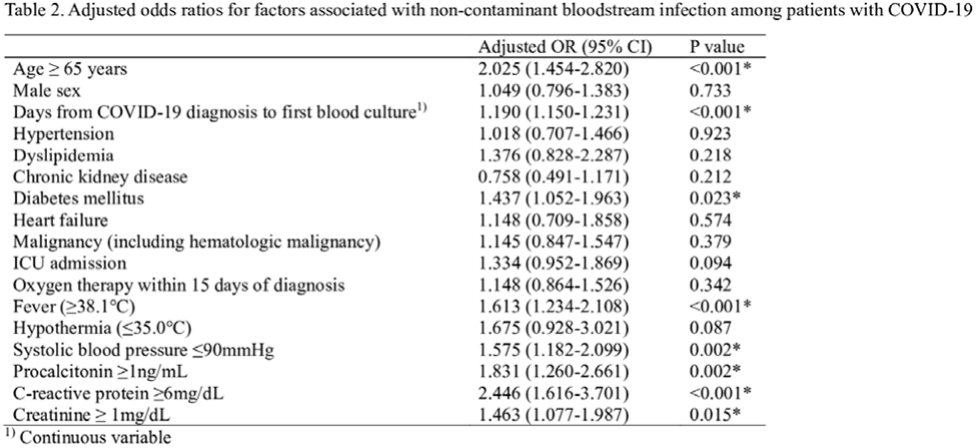

**Results:**

A total of 3,082 hospitalized COVID-19 patients (median age 67 years, 59.1% male) underwent blood culture within 15 days of diagnosis, and 304 (9.9%) had BSI. The number of blood cultures and BSI incidence increased during pandemic surges. BSI was associated with higher 15-day mortality (21.4% vs. 5.0%, p< 0.001). During the study period, a total of 5,095 blood cultures were performed, of which 501 (9.8%) yielded non-contaminant pathogens. Most frequently identified pathogens included *Staphylococcus aureus* (14.4%), *Escherichia coli* (14.2%), *Klebsiella pneumoniae* (12.8%), and *Candida* species (8.2%). In multivariate logistic regression, independent predictors of BSI included age ≥65 years, diabetes, fever ≥38.1°C, systolic BP ≤90 mmHg, procalcitonin ≥1 ng/mL, CRP ≥6 mg/dL, creatinine ≥1.0 mg/dL, and culture ≥2 days after COVID-19 diagnosis.

**Conclusion:**

Identifying clinical predictors for BSI in COVID-19 can guide targeted blood culture practices, improving diagnostic stewardship. Risk-based testing strategies may reduce unnecessary cultures and support efficient resource use in future pandemics.

**Disclosures:**

All Authors: No reported disclosures

